# From Sewage Sludge to the Soil—Transfer of Pharmaceuticals: A Review

**DOI:** 10.3390/ijerph191610246

**Published:** 2022-08-18

**Authors:** Wioleta Bolesta, Marcin Głodniok, Katarzyna Styszko

**Affiliations:** 1 Faculty of Energy and Fuels, AGH University of Science and Technology, Al. Mickiewicza 30, 30-059 Cracow, Poland; 2 Water and Sewage Company in Żory, ul. Wodociągowa 10, 44-240 Zory, Poland; 3 Central Mining Institute, Plac Gwarków 1, 40-166 Katowice, Poland

**Keywords:** pharmaceuticals, sewage sludge, sewage sludge management, fertilizer

## Abstract

Sewage sludge, produced in the process of wastewater treatment and managed for agriculture, poses the risk of disseminating all the pollutants contained in it. It is tested for heavy metals or parasites, but the concentration of pharmaceuticals in the sludge is not controlled. The presence of these micropollutants in sludge is proven and there is no doubt about their negative impact on the environment. The fate of these micropollutants in the soil is a new and important issue that needs to be known to finally assess the safety of the agricultural use of sewage sludge. The article will discuss issues related to the presence of pharmaceuticals in sewage sludge and their physicochemical properties. The changes that pharmaceuticals undergo have a significant impact on living organisms. This is important for the implementation of a circular economy, which fits perfectly into the agricultural use of stabilized sewage sludge. Research should be undertaken that clearly shows that there is no risk from pharmaceuticals or vice versa: they contribute to the strict definition of maximum allowable concentrations in sludge, which will become an additional criterion in the legislation on municipal sewage sludge.

## 1. Introduction

During wastewater treatment, it is not only treated sewage that leaves the treatment plant but also waste, such as screenings, sand, and stabilized sewage sludge, which is created as a result of the continuous multiplication of the biomass of microorganisms. Each of these wastes requires management. The biggest problem is the formation of excessive sewage sludge in large amounts. Its management requires it to undergo previous stabilization processes. According to the currently binding legal acts in Poland, stabilized sewage sludge is a sludge with reduced susceptibility to rotting and does not pose a threat to the environment or human life and health [1,2]. The constant development of cities and, hence, the expansion of sewage networks, causes an increasing amount of sewage [3,4]. Moreover, restrictive standards concerning the maximum permissible concentrations for biogenic elements contribute to the increase in the amount of excess sludge. Statistical data from Statistics Poland confirm the growing amount of sludge in industrial and municipal sewage treatment plants. Since 2010, this value is consistently growing, and in 2019 it exceeded 1048.7 thousand tons of dry matter) [5]. In 2020, there was a decrease in the generated sludge by approx. 5.6% (Table 1). According to the Central Statistical Office, this could be related to the coronavirus pandemic, which forced the human population to be less active which inhibited the industry [5].

Over the last 20 years, the method of sewage sludge management in Poland has changed dynamically. The amount of stored sludge is systematically decreasing, which is the result of the changing law in Poland. In 2000, there were 474.5 thousand tons of dry matter of stored sewage sludge, 10 years later there were 165.9 thousand tons of dry matter, and in 2020 only 63.9 thousand tons of dry matter. A completely opposite tendency is observed when analyzing the thermal treatment of the sludge. In 2000, only 34.1 thousand tons of dry matter were transformed this way, whereas in 2010 the level of thermal transformation almost doubled, and in 2020 it reached the level of 219.4 thousand tons of dry matter. The level of sludge used in agriculture for the cultivation of crops intended for the production of compost remains stable. On the other hand, significantly less sludge is used for land reclamation, including land for agricultural purposes—in 2019 only 24.5 thousand tons of dry matter was managed in this way. The amount of sludge temporarily stored in the sewage treatment plant was also reduced from 14,654 thousand tons to 6143.6 thousand tons of dry matter, which is important from the point of view of safety. Taking into account the agricultural use in total, i.e., applied in agriculture, land reclamation including reclamation of land for agricultural purposes, and the cultivation of plants intended for compost production, it should be noted that, in 2000, as much as 37.77% of all sludge formed were distributed in natural areas. On the other hand, 20 years later, the agricultural use of sludge fell to 21.97%. Undoubtedly, this effect was caused by the introduction of restrictive regulations on pollution caused by sewage sludge.

There is no doubt that the amount of sludge to be managed is enormous and requires special and careful measures. The amount of generated sludge is only about 1–3% of the volume of raw sewage. Nevertheless, they can pose a potential threat to the environment in the event of inappropriate management because they contain, among other things, heavy metals and pathogenic organisms. On the other hand, sewage sludge can be used in practice, as it is a rich source of organic matter and biogenic elements. Sewage sludge is used for agricultural purposes, fertilization of soils and plants as a valuable source of nitrogen and phosphorus, compost production, as well as for the reclamation of degraded lands. Appropriate management of sewage sludge turns out to be important from the point of view of the circular economy, energy economy, and depletion of non-renewable mineral resources.

The article presents a brief overview of the possibilities of sewage sludge management in the context of implementing a circular economy. Particular attention was paid to the agricultural use of sludge. Their use in the natural environment carries the risk of transferring pharmaceutical residues present in sewage sludge and fertilizers produced from them. It is necessary to analyze the content of pharmaceuticals and their fate after the distribution of sludge-based products on farmlands. The aim of the article is to draw attention to the safe usage of sewage sludge in the natural environment in the context of processes involving pharmaceuticals in soil.

## 2. Final Disposal of Sewage Sludge—Granular Fertilizer

It is believed that one of the most beneficial methods of recycling sewage sludge is to use it in agriculture. This is facilitated by the observation of the deficit of organic compounds in soil. The factor limiting the industrial use of sewage sludge in agriculture is the presence of sanitary pollution or the exceeded heavy metal content. Pharmaceuticals are an important problem that may affect the limitation of the use of sewage sludge as fertilizer products, which results from the specificity of their activity and potential possibilities of immunizing pathogenic microorganisms against the active substances contained in the sludge. Application of treated wastewater and digested sludge, and animal manure, are routes of transport of pharmaceuticals of human and veterinary origin to soils and their accumulation. The need for the reduction of our dependence on freshwater irrigation and chemical-based fertilizers coupled with the reuse of wastewater treatment by-products can drive to increase the input of pharmaceuticals in soils in the near future.

Therefore, the article is devoted to the agricultural use of sludge as a fertilizer product. Although there are currently no limits on these substances in sludge, this paper will consider the methodology of testing pharmaceuticals in sludge, and their presence in sewage sludge and soil.

The first patents for the production of fertilizer from sewage sludge are already issued, e.g., in Poland [6]. The process of creating an organic–mineral fertilizer consists of the thorough mixing of stabilized sewage sludge with a dry mass of 18–20% with lime, dolomite, gypsum, and microcrystalline cellulose [7]. The homogeneous mixture obtained in this way is granulated to give the product a suitable, homogeneous granule size and to prevent dusting and excessive disintegration. The last stage of sludge treatment is drying, which additionally protects the product against the possible presence of pathogens.

**Figure 1:**
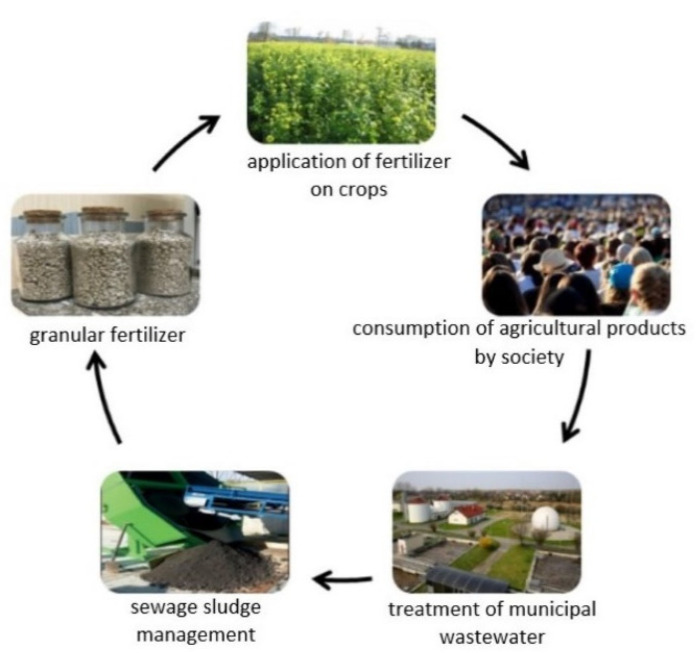
An example of sewage sludge management in a circular economy.

Converting sludge to fertilizer has many advantages. First of all, this process, apparently from all methods of sludge management, is part of the closed cycle trend—the sludge formed in the process of wastewater treatment after modifications returns to the environment as a full-value fertilizer that brings benefits to nature (Figure 1). These benefits result from the high content of phosphorus and nitrogen—biogenic elements necessary for the proper development of plants. The safety of using such fertilizers in agriculture is ensured by removing pathogens and reducing the content of heavy metals. Laboratory tests show that the quality of such products is adequate and meets all the requirements in accordance with the Regulation of the Minister of Agriculture and Rural Development in force in Poland on the implementation of certain provisions of the Act on fertilizers and fertilization [8]. An important aspect is an economic issue. The sludge used in this way does not require a paid collection. On the contrary, it is possible to sell this product as a plant fertilizer rich in minerals.

The field tests which were conducted clearly show the effectiveness of the use of fertilizers made from sludge [9]. An interesting issue, however, is the presence of micropollutants in fertilizing products and the lack of information on the possibility of their spread with fertilization.

## 3. The Content of Sewage Sludge—Properties and Limits in Agricultural Management

The sludge management options are diverse, and the choice depends on factors such as installation costs, operating costs, or the amount of sludge. However, the most important factor is the chemical composition and quality of the sludge that needs to be managed.

Considering the variant of using the sludge for agricultural purposes, e.g., for land reclamation or as an organic and mineral fertilizer, special attention should be devoted to the standards of quality that the sludge must meet. Such sludge management involves the risk of distributing not only valuable ingredients, but also all substances contained in the sludge, including micropollutants, known as the ”emerging contaminants”. It is a wide group of compounds that differ in terms of structure and physicochemical properties. These include the following: pharmaceuticals and their residues, personal care products, repellants, caffeine, bisphenol A, or drugs. The composition of the sludge is variable and individual for each treatment plant. It also depends on the composition of the incoming sewage and the pollutants it contains [10] and the treatment technology [11,12,13].

The stabilized sewage sludge is characterized by high hydration (55–80%) and a high content of organic parts (45–55% dm). The relatively high level of biogenic elements, such as nitrogen, phosphorus, and magnesium, encourages the use of the sludge for agricultural purposes. On the other hand, the presence of heavy metals in the sludge was proven, including arsenic, cadmium, lead, and mercury [14].

Due to the presence of environmentally harmful toxic heavy metals, the sludge intended for agricultural use must meet the standards in the scope of the above-mentioned parameters specified in the relevant legal acts (in Poland: Ordinance of the Minister of the Environment of 6 February 2015 on municipal sewage sludge [15]). In addition, the sludge is tested in the context of the presence of Salmonella bacteria and live eggs of intestinal parasites, such as *Ascaris* sp., *Trichuris* sp., *Toxocara* sp. The sludge dose applied to a specific area of land is also controlled.

In addition, organic–mineral fertilizer based on sewage sludge is also subject to relevant legal acts (in Poland: The Act of 10 July 2007 about fertilizers and fertilization [8]; Regulation of the Minister of Agriculture and Rural Development of 18 June 2008 on the implementation of certain provisions of the Act on fertilizers and fertilization [16]). They define the maximum permissible concentrations of heavy metals: Cr, Cd, Ni, Pb, and Hg. As in the agricultural sewage sludge, the fertilizer is subjected to parasitological analysis.

Meanwhile, the presence of micropollutants or ”emerging contaminants”, such as pharmaceuticals, are identified in the raw sludge, activated sludge, and digested sludge [17]. The content of pharmaceuticals and other micropollutants contained in sewage sludge and fertilizers derived from them has not been standardized so far: it is not recommended to test pharmaceuticals or any reference methods for their determination and compounds requiring specific monitoring are still not identified in sewage sludge [18].

Before steps are taken towards the investment, which is an installation for the production of fertilizers from sewage sludge, its composition should be carefully analyzed in the context of the presence of pharmaceuticals that can be transferred into the soil and have a negative impact on living organisms and the natural environment.

## 4. Analytical Methods for the Determination of Pharmaceuticals

**Figure 2:**
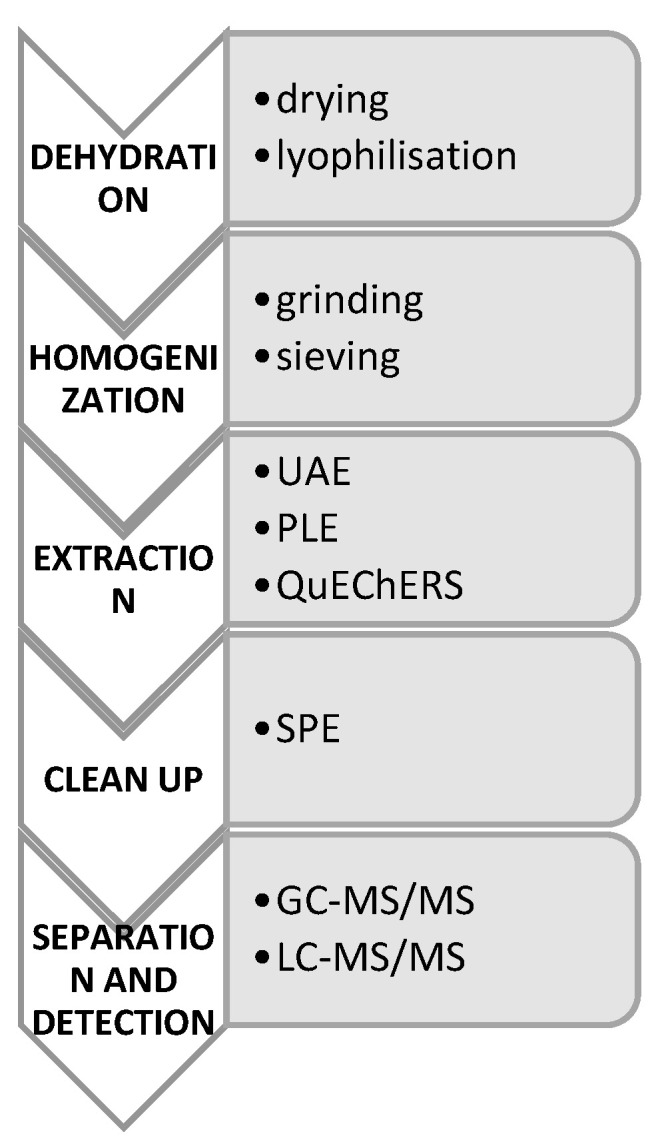
Stages of the analysis of the content of micropollutants in sewage sludge with examples of methods [11,19,20,22]. Abbreviations: UAE—ultrasound-assisted extraction; PLE—pressurised liquid extraction; QuEChERS—quick, easy, cheap, effective, robust, and safe; SPE—solid-phase extraction; GC—gas chromatography; LC—liquid chromatography; MS/MS—tandem mass spectrometry.

Another aspect is the methodology of pharmaceuticals determination in sewage sludge and fertilizers. The analytical method of testing sewage sludge requires sample preparation in several stages. Dehydration, homogenisation, and extraction [19,20,21] are performed (Figure 2). Solid samples (sewage sludge or fertilizer) are frozen and lyophilized. After weighing the milled small aliquot of the sample and sieving about 0.45 µm particles, the pharmaceutical internal determination standard is added. Then, the sample is extracted by ultrasound-assisted extraction (UAE), which is the most commonly used extraction method in 2011–2019 [22,23], or by QuEChERS extraction (quick, easy, cheap, effective, robust, and safe). After centrifugation, the liquid fraction is subjected to a clean-up phase and centrifuged again. The sample is dried and diluted. Due to the non-selectivity of the extraction, it is necessary to clean the sample by SPE (solid-phase extraction) on HLB columns conditioned with methanol and deionized water.

The sample prepared in this way is analyzed using liquid or a gas chromatography e.g., ultra-performance liquid chromatography) system, coupled with mass spectrometry (MS).

The analytical procedure for the determination of pharmaceuticals in sludge is a complex process. The complicated composition of the sample and the complexity of the matrix, makes the choice of the method a difficult task. Very low concentrations of micropollutants make it difficult to determine the compounds within the quantification limits of the method. An additional difficulty has to do with the specificity of the contamination of the analyzed sewage sludge, depending on the composition of sewage in particular seasons and even the size of the city. In order to protect the environment and apply the principles of Green Chemistry, automated methods are developed and implemented that do not require the use of large amounts of reagents (e.g., Quechers).

The obtained results of the content of pharmaceuticals in stabilised sewage sludge ranged from µg/kg to mg/kg dm. The highest concentrations were recorded for antibiotics of the fluoroquinolone group, such as ofloxacin, the content of which was estimated at 8546.21 µg/kg dm [23]. Ciprofloxacin was estimated at 6500 µg/kg dm [24], 3726.8 µg/kg dm [25], and 303 µg/kg dm [26] while norfloxacin was estimated at 2796.68 µg/kg dm [23] and 620 µg/kg dm [24]. High concentrations were obtained for another group of antibiotics—tetracyclines. The oxytetracycline content was determined at the level of 7105.54 µg/kg dm [23] and 742.5 µg/kg dm [27], while the tetracycline content was 4457 µg/kg dm [23]. Equally high values were obtained by analysing bisphenol A, the concentration of which was 92.9 µg/kg dm [28], through 155 µg/kg dm [29] to 3590 µg/kg [30]. Varying results were obtained for hormones. Both low concentrations of 20–40 µg/kg for estrone [31], 5–50 µg/kg for estradiol [31] and 2–20 µg/kg for ethinylestradiol [31], and concentrations below the limit of quantification were obtained [32]. A similar scatter of the results of values was obtained for other studies, in which estrone was not within the detection limits, and the concentration of 17β-estradiol in the stabilized sewage sludge was found at the level of 293.5 µg/kg [33]. Other pharmaceuticals, such as carbamazepine and ibuprofen, were detected at a similar level. The obtained concentrations indicate values ranging from a few to several dozen µg/kg [34].

## 5. Pharmaceuticals in Sewage Sludge

Pharmaceuticals enter the treatment plant together with raw sewage. The sources of these substances are metabolic products of the human body, hospital wastewater, leachate from landfills, animal husbandry, and sewage from the pharmaceutical industry. Wastewater treatment in municipal wastewater treatment plants by the activated sludge method consists of the mechanical separation of solid pollutants, sedimentation of sand and easily deposited suspensions, biological removal of pollutants with the use of microorganisms, and sedimentation of the activated sludge in a secondary sedimentation tank. Such technology does not allow for the reduction in ”emerging contaminants”. There are several possibilities for degradation and micropollutant processes in the environment. This can be the degradation of a substance, the removal of the substance with the treated effluent, or exiting the system on sludge particles. This is a serious problem for the use of sewage sludge in the environment.

The fate of micropollutants in a typical municipal sewage treatment plant depends on their physicochemical properties, such as molecular weight, octanol–water partition coefficient (K_ow_), acid dissociation constant (pKa), solubility, or biodegradability. Other external factors are the sludge treatment method and the operation condition of the wastewater treatment plant [35,36]. It is estimated that more than 70% of relatively hydrophobic contaminants from groups, such as heavy metals, persistent organic pollutants (POPs), brominated flame retardants and some personal care products (PCP), surfactants, plastic additives, hormones, some PCP, some medicines, and household chemicals are usually well disposed of in wastewater treatment plants [37,38]. On the other hand, many more hydrophilic and slightly or moderately biodegradable pollutants, such as some pharmaceuticals, pesticides, and household chemicals (corrosion inhibitors, sweeteners, chelating agents, phosphorus flame retardants) are poorly removed during treatments [37]. In the course of wastewater treatment, pollutants can adsorb on suspended solids, biodegrade or degrade chemically, and are then removed from the water phase [39].

Some pharmaceuticals (e.g., ibuprofen, ketoprofen) will decompose during the wastewater treatment process [38,40,41]. Estrone, sulfamethoxazole, or carbamazepine are difficult to decompose compounds, so they are only partially decomposed and will flow out with the treated sewage [42]. The last way of reducing micropollutants is adsorption on sewage sludge. Non-polar and alkaline compounds, characterized by high hydrophobicity, such as antibiotics such as tetracyclines and fluoroquinolones (e.g., norfloxacin, ofloxacin, ciprofloxacin, and doxycycline), are sorbed on solid particles [22,43]. It is shown that compounds that are components of hormonal agents adsorb well to sediment particles [44,45], which results from their lipophilicity. Settling on the sewage sludge, they leave the aquatic environment and thus do not end up with the treated sewage in the natural environment. This means, however, that these compounds will not be fully degraded, but will only go into the solid phase.

Another aspect is the stabilisation conditions for the sewage sludge. Studies show differences between the use of composting and anaerobic digestion in terms of the concentration of pharmaceuticals. Only 18% of estrogens were removed under aerobic conditions [46]. Meanwhile, a reduction of up to 97–99% of the content of some antibiotics (tetracycline, chlortetracycline) in the sludge after the composting process was observed [47]. One may notice that the temperature of the process is also important. The fermentation process also reduces the content of pharmaceuticals [48,49]. A total of 16 out of 20 tested compounds underwent biotransformation in over 35% [48].

Additionally, in the case of anaerobic stabilization, the dependence of changes in pharmaceuticals with regard to temperature was noted. Degradation of chlortetracycline increased as a result of increasing the temperature of the process [50]. The opposite effect was achieved with estrogens. Their reduction decreased with increasing fermentation temperature [46].

Not all pharmaceuticals are reduced during the stabilisation processes. It has been shown that carbamazepine [46,51] and fluoroquinolones are persistent and resistant to both aerobic and anaerobic biotransformation [52,53]. Doubts arise as to what their future fate will be, which is closely dependent on the method of sewage sludge management.

## 6. The Presence of Pharmaceuticals in Soil after Sewage Sludge Distribution

As mentioned before, studies show that the concentration of pharmaceuticals in soil is strongly correlated with the maturity of the sludge used [54]. The highest concentrations of pharmaceutical compounds were determined only in limed sludge, lower in digested sludge, even lower in composted sludge, and the lowest in solid post-fermentation [54]. At the same time, it is stated that both the aerobic and anaerobic decomposition of sludge stimulate the degradation of pharmaceutical compounds [55]. It is related to the activity of microorganisms. The same processes take place in the soil, this time involving soil bacteria.

The research showed the presence of pharmaceuticals in soil treated with sewage sludge (Table 2). The highest values were recorded for triclosan and triclocarban. Their content reached several thousand ng/g dm. High concentrations of ciprofloxacin, norfloxacin, and ofloxacin were detected in several studies: additionally, these antibiotics were detected after a longer time after the application of the sludge, which indicates the persistence of these compounds in the soil [56,57,58]. Meanwhile, in soil samples treated with compost, the concentrations of this group of antibiotics were definitely lower, and sometimes lower than the LOQ [59].

**Figure 3:**
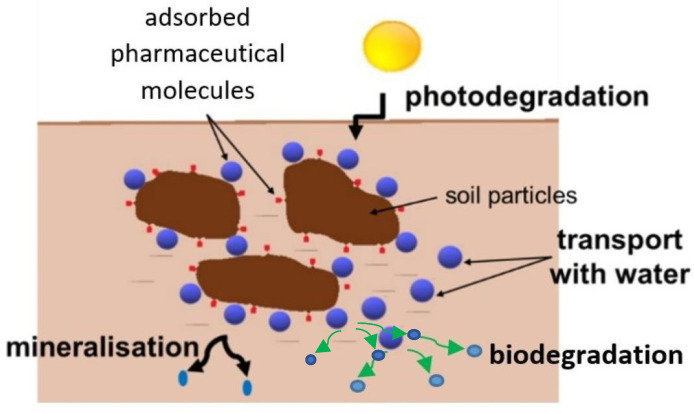
The fate of pharmaceuticals in soil.

The concentration of these compounds depends on the physicochemical properties of the analyzed chemical compound [64] and the different fate of pharmaceuticals in soil, where their content may be reduced due to photodegradation or the mineralisation processes [34,65] (Figure 3).

Studies show that the concentration of pharmaceuticals, such as ibuprofen and ketoprofen, fluoroquinolones (ciprofloxacin and moxifloxacin), and statin atorvastatin decrease under the influence of UV-B radiation [66]. Moreover, research is currently being carried out on the selection of photocatalysts to intensify the process [67,68].

Light radiation naturally contributes to the decomposition of micropollutants, which takes place many times: during the sewage treatment process, sludge oxygen stabilization, sludge composting, and from the moment the sludge is dosed onto the soil.

Pharmaceuticals with strong sorption properties tend to accumulate in the soil, while those which are highly mobile are transported with groundwater and further: to drainage and surface waters [69].

These pharmaceuticals, which are characterized by strong sorption properties, tend to accumulate on soil particles, e.g., trimethoprim, indomethacin, propranolol, metoprolol, and carbamazepine [64]. Other studies shows the presence of pharmaceuticals, such as ciprofloxacin, carbamazepine, ketoprofen, and atenolol in the soil but not in leachate [55]. The results of the studies on diclofenac and carbamazepine confirmed [70,71] significant retention in soil. This was confirmed by other studies [59] showing that norfloxacin, ofloxacin, and ciprofloxacin are very persistent antibiotics and were detected in soil particles, unlike ibuprofen which is detected in soil leachate, which indicates their lower persistence and sorption capacity [59]. The persistence of the pharmaceutical in soil is an important issue. With the passage of time, the concentration of the compound in the soil treated with the sludge is investigated. The results showed different persistence of the soil over the 3-year period of the research: triclocarban, fluoxetine, and diphenhydramine did not show significant changes in their concentration unlike triclosan, which is characterized by a relatively low persistence [58].

The stability of a pharmaceutical will depend on various factors. Studies show that high soil hydration reduces the persistence of clotrimazole, while the opposite will be true of fluconazole in response to soil moisture [72]. The pH of the soil and the content of organic carbon in the soil also has an impact on the durability and rate of decomposition of pharmaceuticals [73]. This is confirmed by other studies based on the enrichment of the soil with fermented sewage sludge and compost rich in organic carbon (OC). OC increases the log K_d_ coefficient, which indicates greater sorption to the fraction with a higher concentration of organic carbon [74,75,76].

Sorption of pharmaceuticals to the soil, apart from the soil pH and organic carbon content, also depends on the mineral parts of the soil [77]. Mechanisms that may affect the adsorption of micropollutants are ion exchange, hydrogen bonding, or the formation of complexes with Ca^2+^, Mg^2+^, Fe^3+^, or Al^3+^ ions [77,78,79]. Hydrophobic interactions, van der Waals interactions, and hydrogen bonds were also proposed, and the pKa coefficients are to help predict the sorption mechanisms of a given compound [80].

The fate of pharmaceuticals in soil and the concentration of pharmaceuticals in soil enriched with sludge will also depend on purely technical conditions. The frequency of sludge application, the amount of sludge dispensed to a specific area, or the time between sludge application and the moment of taking a soil sample will also affect the obtained measurements of pharmaceutical concentrations [81]. The type and physicochemical properties of the soil on which the sludge or the fertilizer based on it are to be applied are also of key importance.

Another aspect is the use of organic and mineral fertilizers based on sewage sludge in agriculture. This variant of sludge management requires separate consideration in terms of the transfer of pharmaceuticals to the soil, because the composition of fertilizers is modified in relation to the composition of the sludge from which it was formed. Considering the changes in the physicochemical composition, the change in pH and other factors influencing the fate of a pharmaceutical, one should expect differences in the transfer of pharmaceuticals from fertilizers to the soil, which is still a completely unknown issue.

## 7. Ecological Effect an Environmental Risk Assessment

Detailed analysis of the dosing of the use of sewage sludge for agricultural purposes is intended to protect the natural environment. The implementation of the use of sludge containing micropollutants in a circular economy carries risks for living organisms.

Agricultural use of sewage sludge affects the quality of soils from areas enriched with sludge and its derivatives [22]. The study of the changing composition of the soil was carried out in relation to the type of applied sludge [54]. These studies showed a relationship between the degree of sludge stabilization and the concentration of pharmaceuticals. In other words, the higher the degree of sludge degradation, the lower the level of pharmaceutical substances was recorded in the soil treated with the sludge [54]. In addition, there was a lower level of leaching of pharmaceuticals into the soil. This indicates that a high level of sludge stabilization may determine the safety of sewage sludge use [54].

The presence of pharmaceuticals was also analyzed in the context of the effect on microorganisms. Antibiotic resistance is a phenomenon that may be intensified due to the contact of antibiotics with soil bacteria, as a result of treating the soil with sewage sludge [82,83]. This is confirmed by other studies that show that the short-term effect of antibiotics on microorganisms has an inhibitory effect. After prolonged exposure to drugs, the activity and biomass of the bacteria return to the level before the tests [84].

The first watch List of substances for union-wide monitoring in the field of water policy was defined in the Decision 2015/495/EU (EC, 2015) and, recently, a third watch list was proposed by the European Union in the Decision (EU) 2022/130 of 22 July 2022 [85]. The presence of antibiotics on the watch lists confirms their direct risk to human and animal health and careful monitoring is recommended by the EU Member States. The watch list also supports the use of it to improve knowledge of the occurrence and spread of antimicrobials in the environment. Communication from the Commission to the European Parliament, the Council and the European Economic and Social Committee published in March 2019 performed the European Union strategic approach to pharmaceuticals in the environment [86]. It highlights that more information is still needed to understand and evaluate certain pharmaceuticals as regards their environmental concentrations and the resulting levels of risk.

Some of the antibiotics we use end up in sewage sludge, together with a variety of antibiotic resistant bacteria present in feces [87,88]. Therefore, there is a widespread concern that spreading sludge on farmland would contribute to the development or spread of antibiotic resistance [89,90]. Antibiotics can accumulate in food webs and, even more alarmingly, antibiotic resistance genes can be transferred between environmental bacteria and human pathogens [91]. Antibiotics and their effects on the environment have become an important theme in environmental science. Sewage sludge is the most important output from WWTPs, and its treatment, reuse, and disposal are the most complex problem [92].

It is proven that plants can accumulate micropollutants [93,94]. Pharmaceuticals reach plant organisms through diffusion and transpiration. These substances accumulate in the roots, e.g., triclosan or carbamazepine, and even in the stems and leaves, e.g., diclofenac, propranolol or chloramphenicol [95]. As the research on the accumulation of pharmaceuticals in spinach shows [96], it cannot be clearly stated what the fate of a given pharmaceutical in contact with the cultivation of a given plant will be because external factors, such as soil type and pH, will be of great importance.

The water cycle in nature causes contaminants from soils to be washed into the water flowing through it and, together with groundwater, get into aquatic environments, and even into waters intended for consumption. The scientific literature proves this negative influence [97]. Hormonal compounds, which are components of drugs regulating the hormonal balance, disturb the reproduction of small aquatic organisms [98]. Other organisms living in the aquatic environment under the influence of endocrine compounds have shown feminization of males through the loss of male features [99]. Bisphenol A also affects the reproductive system of living organisms [100]; therefore, it is classified as an endocrine disrupting compound that is important for species reproduction. Anti-inflammatory drugs also have negative effects, e.g., ibuprofen, diclofenac, and E2 pose chronic risks for high trophic level organisms [101].

To assess the potential ecotoxicological effect of a test compound, the substance risk ratio (RQ) and the ratio between the measured (MEC) or predicted environmental concentration (PEC) and predicted no effectcConcentrations (PNEC) are determined [102,103]. A very simple classification for risk assessment by RQ divides substances with low (RQ < 0.1), medium (RQ between 0.1–1), and high risk (RQ > 1) environmental effects [104]. It was also proposed to assess the environmental risk in soil based on pharmaceutical concentrations and PEC_soil_ calculation from the formula of the European Commission Technical Guidance Document on Risk Assessment [105]:

PEC_soil_ = C_sludge_ × APPL_sludge_/DEPTH_soil_ × RHO_soil_

where C_sludge_ is the concentration of pollutants in the sludge; APPL_sludge_ is the dry sludge application rate (0.5 kg/m^2^ year); DEPTH_soil_ is the depth of soil mixing (0.20 m); and RHO_soil_ is the bulk density of wet soil (1700 kg/m^3^) [33].

## 8. Conclusions

Due to the content of pharmaceuticals in the sewage sludge, their ecological management requires a multifaceted analysis. The physicochemical diversity of these compounds contributes to the different processes that pharmaceuticals undergo. The quality and composition of soil enriched with sewage sludge will also affect the fate of micropollutants.

This article shows that pharmaceuticals that enter the soil with sludge can decompose, be diluted and transported with water to further lands, or accumulate on soil particles. This means a real threat to organisms whose natural habitat is soil, as well as to humans and animals whose food may be planted in areas enriched with sludge. Most of all, the quality of the water that circulates in nature is at stake.

Additional detailed analyses of the fate of pharmaceuticals in the soil should be undertaken if sewage sludge is to be safely implemented as part of a circular economy and used for agricultural purposes. This issue is still poorly understood because of the large number of factors influencing the processes taking place. The type of micropollutants in any given sludge should be determined, as well as the soil composition, and a detailed method of dosing the sewage sludge, i.e., the amount and frequency of dosing and possibly the weather conditions.

Other aspects have to do with the use of fertilizers based on sewage sludge. Their properties are changed due to the changed components of sludge. Physicochemical changes will undoubtedly affect the transfer of pharmaceuticals to the soil; therefore, separate tests should be undertaken to obtain information on the behaviour of micropollutants in the soil introduced with the fertilizer.

## Figures and Tables

**Table 1:** Total production of sewage sludge from industrial and municipal wastewater treatment plants and trends in their use over the last 20 years [5].

Specification	2000	2005	2010	2015	2019	2020
	in Thousand Tons of Dry Solid					
Total sewage sludge generated during the year of which:	1063.1	1124.4	895.1	951.5	1048.7	989.5
applied in agriculture;	212.2	98.2	136.9	126.6	141.9	160.4
applied in land reclamation including reclamation of land for agricultural purposes;	154.9	324.9	150.4	31.3	24.5	26.5
applied in cultivation of plants intended for compost production;	28.1	29.6	31.3	48.2	31.7	30.5
thermally transformed;	34.1	37.4	66.4	165.4	195.7	219.4
landfilled;	474.5	399.1	165.9	131.5	113.3	63.9
sewage sludge accumulated on the wastewater treatment plants	14,654	9342.8	6450.5	6483.9	6191.2	6143.6

**Table 2:** Concentration of pharmaceuticals in soil treated with sewage sludge.

Compound	Measured Concentration [ng/g dm]	References
Ciprofloxacin	350–400 after 8 months of sludge application	[56]
	270–280 after 21 months	[56]
	450 (2.5 cm depth)	[57]
	542 (day 0)–390 (day 994)	[58]
	<LOQ–8.7 (soil amended with composted sludge)	[59]
Norfloxacin	320–290 after 8 months of sludge application	[56]
	270–300 after 21 months of application	[56]
	350 (2.5 cm depth)	[57]
	50 (day 0 in a mesocosms experiment)	[58]
	<LOQ–9.4 (soil amended with composted sludge)	[59]
Ofloxacin	470 (day 0)–267 (day 994)	[58]
	5.3–8.6 (soil amended with composted sludge)	[59]
Triclosan	1715 (day 0)	[58]
	833	[60]
	10,900	[61]
	14,000	[62]
	n.d.–16.7	[63]
Triclocarban	2715	[58]
	4940	[61]
	8000	[62]
Trimethoprim	n.d.–0.64	[60]
	n.d.–60.1	[63]
Azithromycin	30 (day 0 in mesocosms experiment)	[58]
Diclofenac	n.d.–1.16	[63]
Ibuprofen	n.d.–5.03	[63]
	63.5	[61]
	750	[62]
Carbamazepine	n.d.	[60]
	0.02–7.5	[63]
	6 (day 0 in a mesocosms experiment)	[58]
	9	[62]
	183	[61]
Fluoxetine	10 (day 0 in a mesocosms experiment)	[58]
Diphenhydramine	40 (day 0 in a mesocosms experiment)	[58]
	n.d.	[60]

n.d.—not detected.

## Data Availability

Not applicable.
